# Serum selenium levels of pre-eclamptic and normal pregnant women in Nigeria: A comparative study

**DOI:** 10.1371/journal.pone.0238263

**Published:** 2020-08-27

**Authors:** Stephen Chijioke Eze, Nathan Azubuike Ododo, Emmanuel Onyebuchi Ugwu, Joseph Tochukwu Enebe, Onyema Athanatius Onyegbule, Innocent Okafor Eze, Bamidele Uche Ezem

**Affiliations:** 1 Department of Obstetrics & Gynaecology, Federal Medical Centre (FMC), Owerri, Nigeria; 2 Department of Obstetrics and Gynaecology, Faculty of Medical Sciences, College of Medicine, University of Nigeria, Ituku-Ozalla, Enugu, Nigeria; 3 Department of Obstetrics & Gynaecology, Enugu State University of Science and Technology College of Medicine/Teaching Hospital, Parklane, Enugu, Nigeria; 4 Departments of Obstetrics and Gynaecology, Faculty of Medical Sciences, College of Medicine, Imo State University, Orlu, Imo State, Nigeria; Texas A&M University College Station, UNITED STATES

## Abstract

**Introduction:**

Trace element selenium, an antioxidant, and peroxynitrite scavenger when incorporated into selenoproteins and enzymes reduce oxidative stress which is implicated in the aetiopathogenesis of pre-eclampsia. A paucity of information exists on the serum selenium levels among pre-eclamptic pregnant women in Nigeria, hence the need for this study.

**Objective:**

To compare mean serum selenium levels and prevalence of selenium deficiency in preeclamptic pregnant women and their normotensive pregnant controls.

**Materials and methods:**

A comparative case-control study was carried out at the Department of Obstetrics and Gynaecology, Federal Medical Centre, Owerri, Imo state. Fifty-eight preeclamptic and equal normotensive pregnant controls were matched for age groups, gestational age groups, parity groups, and socio-economic status had their serum samples analyzed for selenium level using atomic absorption spectrophotometer (ASS). Data analysis was done using the statistical package for social sciences (SPSS) version 20.0. P-value of < 0.05 was considered to be statistically significant.

**Result:**

Mean serum selenium levels of the preeclamptic women(0.67±0.27μmol/l) was significantly (p<0.001) lower than that of the normotensive controls(1.20±0.46μmol/l). Selenium deficiency occurred significantly more in preeclamptic women (33(56.9%) than normotensive women (10(17.2%). Pearson’s coefficient analysis showed negative correlation between serum selenium level with severity of systolic blood pressure (Correlation Coefficient (r) = -0.593), diastolic blood pressure(r = -0.519) and severity of preeclampsia(r = -0.598).

**Conclusion:**

Serum selenium levels of pre-eclamptic women were significantly lower compared to that of normotensive pregnant controls and selenium deficiency occurred significantly more among the preeclamptic pregnant women compared to the normotensive controls. Selenium level dynamics in pregnancy possibly could play a role in the incidence of pre-eclampsia among pregnant women.

## Introduction

Preeclampsia is a transient hypertensive disorder of pregnancy that could lead to fatal conditions. High mortality and perinatal morbidity are associated with this disease of pregnancy and it affects approximately 5–10% of pregnancies worldwide [1,2]. It is the third most common cause of maternal death worldwide [3,4]. Developing countries are more adversely affected as 20–80% of increased maternal mortality is associated with pre-eclampsia while in developed countries it has contributed to a five-fold increase in perinatal mortality responsible for 15% of preterm births [5]. Presently this disease cannot be cured and it usually leads to pre-term caesarean delivery. In Nigeria, preeclampsia and eclampsia contribute 10–20% of all maternal deaths [3,4,6].

Despite several studies on preeclampsia, its exact aetiology remains poorly understood. However, placental defects and oxidative stress have been implicated and observed to develop early in pregnancies affected by the disease [7,8]. Also, the maternal circulation and the placenta were reported to have increased levels of markers of oxidative stress in women with pre-eclampsia [9]. Some antioxidant supplements (vitamin C & E) have been shown to have the capacity to reduce the incidence of preeclampsia in women at high risk (previous history of preeclampsia, the occurrence of pre-eclampsia in first-degree relatives, and pre-existing diabetes, hypertension, thrombophilia, obesity, and renal disease) of developing the condition [10]. Oxidative stress exists when the body’s system ability to neutralize and excrete free radicals and other active intermediates generated in it is exceeded by the production of these radicals. Placental and maternal systemic oxidative stresses lead to a generalized inflammatory process that occurs in pre-eclampsia and it is a major event in this multi-systemic disorder [11,12].

Besides, poor placental formation is one of the early preclinical events in pre-eclampsia and this leads to reduced perfusion of the placenta producing reactive oxygen species that result in ischaemic-perfusion injury. Consequently, generalized maternal inflammatory response results and causes generalized endothelial cell dysfunction which produces the characteristic symptoms of hypertension, proteinuria and sudden oedema [13]. However, the placenta itself harbours some anti-oxidant defences, including the selenium-dependent glutathione peroxidases, thioredoxinreductases, selenoprotein-P, and copper/zinc and manganese superoxide dismutases (Cu/Zn and Mn SODs), which prevents the placenta from necessary injury. These anti-oxidant enzymes are rich in trace elements for their optimal functions. This is the link between trace elements and other anti-oxidants with the pathogenesis of preeclampsia.

Despite several studies on preeclampsia, the exact aetiology remains largely unknown. Selenium is a component of some of the antioxidant enzymes and proteins harboured by the body and placenta, and Selenium is implicated in the placental protection against oxidative stress, one of the hallmarks of preeclampsia [8,14].

There has been an inconsistency (decreased selenium levels and no significant difference in selenium levels in normal pregnancy and pre-eclamptic women) in the relationship between the levels of serum Selenium and the occurrence of pre-eclampsia [15–17]. There is a scarcity of studies concerning maternal selenium status during pregnancy in Nigeria and inconsistency in findings from published studies led to the development of this research. This study will help determine a baseline for selenium levels among pregnant women in the study area and may also contribute to the existing body of knowledge on the role of Selenium in the pathogenesis of preeclampsia.

## Materials and methods

### Study area and population

This study was a case-control study that was conducted in the Maternity Unit of the Department of Obstetrics and Gynaecology of the Federal Medical Centre, Owerri, Imo State, South-East Nigeria. The study was conducted over 2 years between November 2014 and November 2016. The study population were pregnant women with a diagnosis of preeclampsia (cases) and women with normal pregnancies (controls). Only women with singleton pregnancies were recruited for this study. Pregnant women with chronic illnesses such as human immunodeficiency virus (HIV) infection, chronic renal disease, and diabetes mellitus were excluded from this study. Also excluded were pregnant women with multiple gestations and those on medications that could affect their serum selenium levels e.g, supplements containing selenium and those that smoked or took alcoholic beverages.

### Sampling technique

Consecutive eligible, consenting preeclamptic women were enrolled from the antenatal clinic and labour ward. After selecting a study participant, an eligible consenting normotensive pregnant woman matched for age, gestational age, parity, and socioeconomic status were enrolled from the same population to serve as controls and this selection process was continued until the sample size for the study was reached.

### Sample size calculation

Calculated sample size for each group (n) was 52 using the formula n = 2Z^2^PQ/d^2^ using 95% confidence interval with 0.05 precision and assuming a 10% attrition rate. A prevalence of 1.7% of preeclampsia in Nnewi a neighbouring town to Owerri as reported by Mbachu et al. [18] was utilized. A total of 116 pregnant women were therefore recruited for both groups in this study.

### Ethical considerations

Ethical clearance FMC/OW/HREC/29 was obtained from the Ethics and Research Committee of the Federal Medical Centre, Owerri. Written Informed consent was also obtained from each participant that was recruited for the study after adequate individualized counselling. They were assured of the confidentiality of the information obtained from them and that the research could improve the management of women with preeclampsia.

Consecutive eligible, consenting preeclamptic women were enrolled from the antenatal clinic and labour ward. Eligible consenting normotensive pregnant women matched for age-groups, gestational age groups, parity groups, and socioeconomic status were subsequently recruited.

### Sample collection and biochemical analysis

Blood samples (5mls) used for this study were collected through a venepuncture made on the convenient arm of the patient preferably on the veins overlying the cubital fossa. The sample collected was left to stand for fifteen minutes for clot retraction to take place. The sample was spun at 3000 rpm for 10 minutes and the supernatant collected was stored in a freezer at -20°C until analysis. The analysis was carried out by the laboratory scientists at the Project Development Institute (PRODA), Enugu in conjunction with the investigator. The samples were transported from FMC, Owerri (study centre) to PRODA, Enugu in a cold-chain box with ice packs. The laboratory scientist was blinded throughout the analysis. The level of selenium was estimated using flame atomic absorption spectrophotometry (AAS). Quality control was ensured throughout the analysis of the samples by the use of commercially prepared samples that ensured that the same sensitivity and specificity were maintained throughout the analysis. Atomic absorption spectrophotometer model 210VGP (Buck Scientific, USA) was utilized for the analysis of the samples.

### Statistical analysis

Data collected was analyzed using the Statistical Package for Social Sciences (SPSS) software for windows version 20.0(SPSS Inc., Chicago, IL). Proportions were compared with Pearson Chi-square for categorical variables while means were compared using students t-test. Data were presented using tables and charts as appropriate. Values were set at 95% confidence level, a P-value of 0.05 was considered to be significant.

Concerning this study, deficiency states of selenium for the participants was defined as serum values less than the lower level of the normal range of selenium in an adult human [0.8–2.0(μmol/l)] [19].

## Results

### Basic characteristics of participants

The preeclamptic patients and the normotensive controls were matched for age, socioeconomic status, parity, and gestational age. It was observed in the study that many (37.9%) of each group of the participants were aged 25–29 years and only a few of the participants were recruited at the extremes of age. Only two (3.5%) of each of the cases and controls were 40 years and above while the majority of both groups were of the high and middle socioeconomic classes.

In the study, 36.2% of the preeclamptic women were unbooked which was more than 10.3% noted in the normotensive group. There were more (41.4%) nulliparous women in the two groups than primipara and higher parities. The majority (72.4%) of the participants were at the gestational age of 34 weeks and beyond. The details of the basic characteristics are shown in Tables 1 and 2

**Table 1:** Basic characteristics of participants.

*Variable*	*Control*		*Preeclamptic*		*P-value*
	N	(%)	N	(%)	
***Age in Years***					1.000
<*20*	0	0	0	0	
*20*–*24*	2	3.45	2	3.45	
*25*–*29*	22	37.9	22	37.9	
*30*–*34*	19	32.8	19	32.8	
*35*–*39*	13	22.4	13	22.4	
>*40*	2	3.45	2	3.45	
***Social class***					1.000
*Upper*	20	34.5	20	34.5	
*Middle*	27	46.6	27	46.6	
*Lower*	11	19	11	19	
***Maternal weight in kg***					0.051
<*90*	43	74.1	33	56.9	
≥*90*	15	25.9	25	43.1	
***Body Mass Index in kg/m2***					0.396
<*18*.*5*	0	0	0	0	
*18*.*5*–*24*.*9*	7	12.1	6	10.3	
*25*–*29*.*9*	20	34.5	20	34.5	
*30*–*34*.*9*	21	36.2	15	25.9	
*35*–*39*.*9*	8	13.8	10	17.2	
≥*40*	2	3.4	7	12.1	
***Booking Status***					
*Booked*	52	89.7	37	63.8	0.001
*Unbooked*	6	10.3	21	36.2	
***Parity***					1.000
*Nullipara*	24	41.4	24	41.4	
*Primipara*	10	17.7	10	17.7	
*Multipara*	22	37.9	22	37.9	
*Grandmultipara*	2	3.4	2	3.4	
***Gestational Age in Weeks***					1.000
<*34*	16	27.6	16	27.6	
≥*34*	42	72.4	42	72.4	

**Table 2:** Comparison of the clinical characteristics between cases and control.

*Variables*	*Controls Mean ±SD*	*Preeclamptic Mean ± SD*	*t-test*	*P-value*
***Age in Years***	31.45 ± 4.9	31.03 ± 4.73	0.463	0.644
***BMI in kg/m***^***2***^	30.40 ± 4.47	31.70 ± 6.00	-1.326	0.188
***GA in weeks***	36.36 ± 3.65	35.22 ± 4.88	1.422	0.158
***SBP in mmHg***	108.53 ± 11.08	170.43 ± 22.23	-18.979	<0.001
***DBP in mmHg***	68.88 ± 8.84	107.33 ± 15.11	-16.729	<0.001

BMI = Body mass index, GA = Gestational age, SBP = Systolic blood pressure, DBP = Diastolic blood pressure.

### Comparison of mean serum selenium level in normotensive and preeclamptic women

The mean serum selenium level in the preeclamptic group (0.67±0.27 μmol/l) was lower than that of the normotensive control (1.20±0.46μmol/l). The observed difference was statistically significant (P<0.001) as shown in Table 3.

**Table 3:** Comparison of mean serum selenium levels in the normotensive and preeclamptic women.

*Selenium*		*Normotensive (n = 58)*	*preeclamptic (n = 58)*	*t-test*	*P value*	*OR (CI = 95%)*
		Mean±SD	Mean±SD			
***Se Serum*(*μmol*/*L*)**		1.20±0.46	0.67±0.27	7.538	<0.001	0.388–0.666

*Se = Selenium.

### Comparison of selenium deficiency in normotensive and preeclamptic women

**Fig 1:**
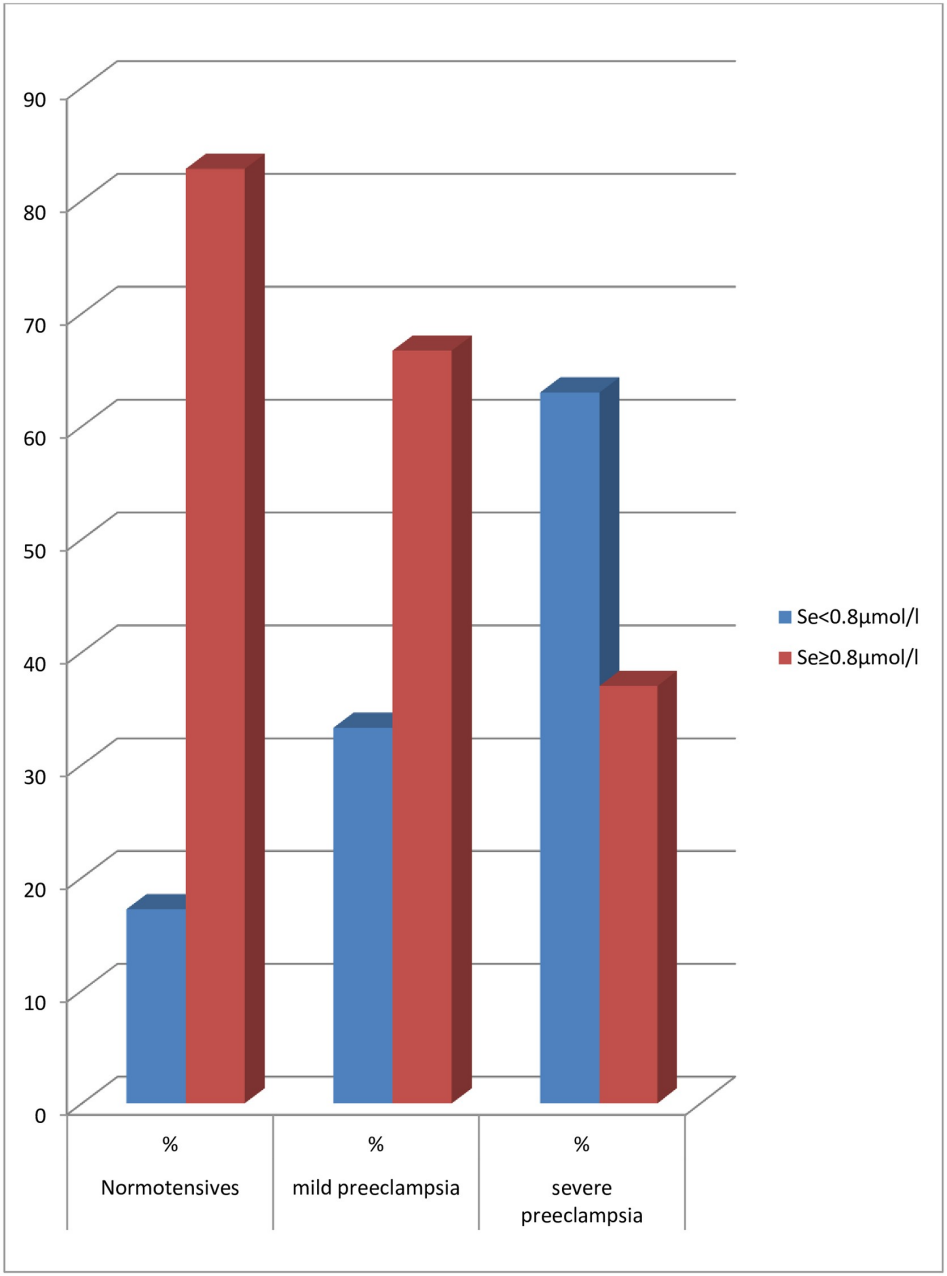
The prevalence of selenium deficiency among normotensive, mild and severe preeclamptic women.

The proportion of participants in each arm of the groups that had serum selenium level below the normal value of selenium (selenium deficiency) were shown in Table 4. It was revealed that 56.9% of the preeclamptic group compared to 17.2% of normotensive controls had selenium deficiency. The observed difference in proportion was statistically significant (P<0.001). When the preeclamptic group was subcategorized into mild and severe preeclamptic groups, it was further shown that among the preeclamptic group, 33% of those with mild preeclampsia and 63% of the severe preeclampsia had selenium deficiency as shown in Fig 1.

**Table 4:** Comparison of selenium deficiency in preeclamptic and normal pregnant women.

*Selenium Deficiency*	*Normotensives N (%)*	*Preeclamptic N (%)*	*P-value*	*OR (CI 95%)*
***Yes***	10(17.2)	33(56.9)	<0.0.001	0.158 (0.067–0.372)
***No***	48(82.8)	25(43.3)		

### The relationship between the mean serum level of selenium and the severity of preeclampsia

It was observed that 20.7% of the preeclamptic women had a mild disease while 79.3% had severe disease. The mean serum selenium level in the latter group (0.62± 0.25μmol/l) was observed to be significantly (P<0.03) lower than the former group (0.87± 0.25μmol/l) as was shown in Table 5.

**Table 5:** Comparison of mean Selenium level between the mild and the severe preeclamptic women.

Preeclampsia	N	%	Mean ± SD	P-value
**Mild**	12	20.7	0.87 ± 0.25	0.03
**Severe**	46	79.3	0.62 ± 0.25	

**Fig 2:**
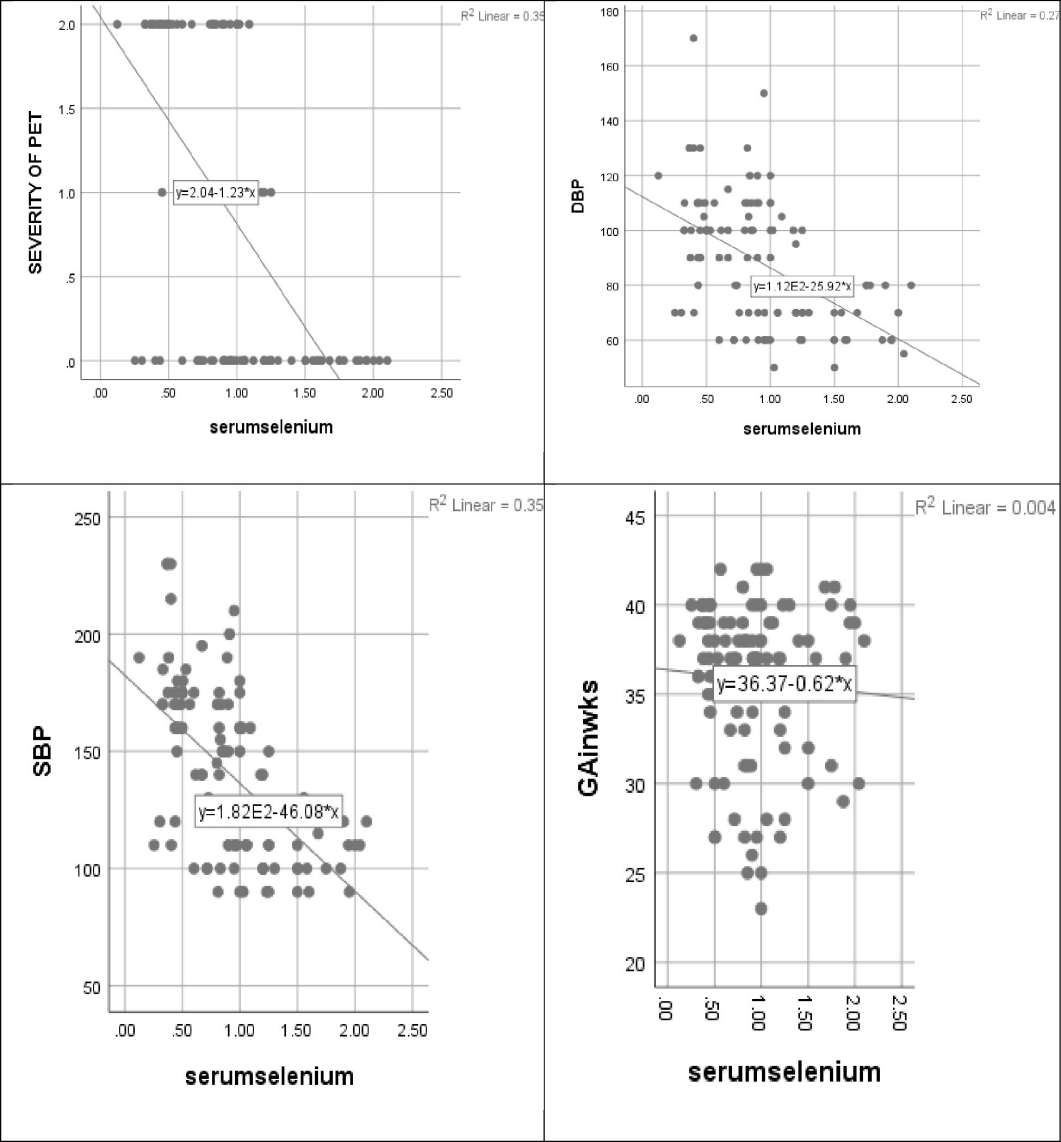
(a), (b), (c) and (d) represent the correlation of serum selenium concentration with the severity of preeclampsia, diastolic BP, systolic BP, and gestational age respectively.

Pearson’s correlation analysis was done to show the relationship between variables: serum selenium, severity of preeclampsia, systolic and diastolic blood pressure, and gestational age. There was an inverse relationship between serum selenium and severity of preeclampsia (r = -0.598, p<0.001), DBP (r = -0.519, p<0.001), and SBP (r = -0.593, p<0.001) while there was no significant relationship between serum selenium concentration and GA (r = -0.065, p = 0.485) (r = correlation coefficient, GA = gestational age, SBP = systolic blood pressure, DBP = diastolic blood pressure). The details are as shown in Fig 2.

## Discussion

The mean selenium level of the participants in this study was comparable to that of Akinloye et al. [20] in Oshogbo, Nigeria. This study also reported a statistically significantly lower mean selenium level in preeclamptic women compared to normotensive pregnant controls. This finding is also in consonance with the findings of Akinloye et al. [20] in Oshogbo South-West Nigeria. These lower selenium levels were also shown in many other similar studies [16, 21–24]. Farzin et al. [25] in Iran found a significantly lower selenium level between the preeclamptic and normotensive pregnant women, although serum selenium levels were within the normal range in both groups. Also, other similar studies by Negi et al. [21] in India and Haque et al. [22] in Bangladesh obtained similar findings (reduced selenium levels) in serum selenium levels of preeclamptic and normotensive women. However, our findings differed from the findings of Rayman et al. [17] who in a case-control study in Southern Brazilian study found no statistical difference between the reduced serum selenium concentration of both the pre-eclamptic and normal pregnant women.

Importantly, this decrease in selenium level as a cause or an effect of preeclampsia is not certain. Selenium is a component of the antioxidant enzymes and selenoproteins. Increased oxidative stress in women with preeclampsia has been demonstrated in earlier studies [26,27]. The decrease in serum selenium observed among the preeclamptic group in this study may be connected to the role selenium plays as an antioxidant and subsequently the development of pre-eclampsia in women with low levels of selenium.

Furthermore, differences in the report from different regions of the world call for caution in the interpretation of the results of serum selenium levels in studies. The dietary intake as well as the ethnicity of the individual should be considered. Further studies may need to be done to determine the critical level of serum selenium below which preeclampsia is likely to occur. On the other hand, this study found that the mean selenium level of the normotensive controls was 1.20 ±0.46μmol/L and this value falls within the normal range of serum selenium. This is consistent with the findings of Nwagha et al. [28] in a similar study in Enugu, Nigeria. This may suggest that the selenium level of pregnant women in South-East Nigeria is likely to be adequate however large population studies may be needed to confirm this.

On another aspect, Selenium deficiency among the participants in this study was recorded in 17.2% and 56.9% of the normotensive and preeclamptic women respectively. Among the preeclamptic women, 33% of those with mild disease and 63% of those with severe preeclampsia had selenium deficiency. Nutritional deficiency is a common finding in resource-poor countries like Nigeria, and this may have contributed to the prevalence of 17.2% of selenium deficiency noted among normotensive pregnant controls. Kassu et al. [23] reported a prevalence of 18.9% in normotensive pregnant women in Ethiopia and this is similar to that recorded in this study. Selenium deficiency was more prevalent among preeclamptic women compared to normal control in this study. This was consistent with the report of an earlier investigation in Nellore, India by Kaliki et al. [24] They reported that 62% of the preeclamptic women had a serum selenium level of less than 50μg/l (<0.633μmol/l). However, this finding contrasts that of Oguizu [29], which reported no case of selenium deficiency among the studied population in Obiakpor LGA of River State, Nigeria.

The deficiency of micronutrients contributes significantly to the development of preeclampsia. Selenium is an essential component of various selenoenzymes, which are involved in the defence against oxidative stress thus avoiding damage to the endothelial cells caused by reactive oxygen species. The deficiency of Selenium has been associated with hypertensive diseases in pregnancy [30,31]. A positive correlation between increasing selenium concentration and lower incidence of preeclampsia was reported by Vanderlelie et al. [32] in a systematic review of peer-reviewed articles. Some studies have shown that selenium supplementation in pregnant women was associated with a reduction in the incidence of preeclampsia [33,34]. Therefore, serum selenium deficiency that is more prevalent in the preeclamptic group may suggest that selenium deficiency is a possible risk factor for the development of preeclampsia.

Although serum selenium deficiency was found to increase with increasing severity of preeclampsia, other factors like time of onset of the disease, presence or absence of co-morbidities and the degree of response of the arteries to vasoconstrictive and vasodilatory factors released in preeclampsia equally play a role in determining the severity of preeclampsia. This finding implies that the low selenium level found in preeclamptic women cannot only be responsible for the severity of preeclampsia noted among the participants. Among the study participants, the mean selenium levels of participants with severe pre-eclampsia were observed to be lower (0.62±0.25μmol/L) than in those with mild pre-eclampsia (0.87±0.25μmol/L). Kaliki et al. [24] in a case-control study in India reported a similar result. This further still suggests that low selenium may play a role in the aetiopathogenesis of preeclampsia.

In addition, Pearson’s product-moment correlation analysis carried out to determine the relationship between variables, noted a statistically significant (p<0.001) negative correlation between serum selenium levels and severity of preeclampsia indicating that the lower the level of serum selenium, the more the severity of the disease. Pearson’s correlation coefficient (r) of -0.598 indicates a good association between serum selenium and preeclampsia. However, this does not imply causality. Also, serum selenium was found to be significantly (p<0.001) negatively correlated to systolic and diastolic blood pressures (r = -0.593 and r = -0.519 respectively). This is not surprising since both systolic and diastolic blood pressures were used to define the severity of preeclampsia. Other studies also reported similar findings [20,22]. Dhanajaya et al. [35], in an Indian study, found a negative correlation between oxidative stress and the severity of preeclampsia. Serum selenium was negatively correlated to gestational age and this is consistent with earlier findings of some previous studies [22,28,36], however, Karita et al. [37] reported no significant difference in the selenium level among pregnant Japanese women according to pregnancy trimesters. The finding implies that as gestational age increases serum selenium decreases. Hemodilution of pregnancy and an increase in demand for Selenium by the developing fetus may be responsible for this finding. However, increasing oxidative stress may play a role. This is supported by the finding of a positive correlation between oxidative stress and gestational age and a negative correlation between total antioxidant capacity and gestational age in previous studies [38–40].

### Strengths of study and future research direction

A large sample size was used in this study and one of the latest technologies in the analysis of trace elements was utilized in the analysis of Selenium in the samples. A multicentre study will be needed to find the true level selenium among pre-eclamptic and normal pregnant women in Nigeria.

### Limitations

This study was hospital-based and as such may have entertained some level of selection bias. The hospital-based nature of the study could also limit the generalization of the result findings to the entire population.

## Conclusion

This study established that the mean level of selenium was significantly lower among pre-eclamptic pregnant women compared to their normotensive pregnant counterparts and that these low selenium levels among the pre-eclamptic participants worsened with increasing severity of the disease. This research also observed a high prevalence of selenium deficiency in preeclamptic women and this deficiency worsened with the increasing severity of the disease. Selenium deficiency may, therefore, have a role in the aetiopathogenesis of preeclampsia.

## Supporting information

S1 File Serum Selenium in pregnant women in FMC, Owerri, Nigeria. (SAV)
